# Diagnostic accuracy of the vestibular activities and participation measure subscales to detect severe handicap in individuals with vestibular dysfunction

**DOI:** 10.1177/09574271261446597

**Published:** 2026-06-23

**Authors:** Maria das Graças de Araújo Lira, Lucas Barbosa de Araújo, Juliana Cirilo Soares de Souza, Susan L. Whitney, Karyna Myrelly Oliveira Bezerra de Figueiredo Ribeiro

**Affiliations:** 1 Graduate Program of Physical Therapy, 28123 Universidade Federal do Rio Grande do Norte, Natal, Rio Grande do Norte, Brazil; 2 Department of Physical Therapy, University of Pittsburgh, Pittsburgh, PA, USA; 3 Department of Physical Therapy, 28123 Universidade Federal do Rio Grande do Norte, Natal, Rio Grande do Norte, Brazil

**Keywords:** dizziness, predictive value of tests, data accuracy, sensitivity and specificity

## Abstract

**Introduction:**

The subscales of the Vestibular Activities and Participation measure (VAP) assess the impact of vestibular dysfunction on activity and participation; however, no diagnostic accuracy studies have evaluated their ability to identify severe handicap in individuals with vestibular disorders.

**Objective:**

To evaluate the diagnostic accuracy of the VAP subscales in detecting severe handicap in individuals with vestibular dysfunction.

**Methods:**

The diagnostic accuracy study included individuals with vestibular dysfunction who were ≥18 years. The diagnostic accuracy of the VAP subscales (VAP1-BR and VAP2-BR) was analyzed using Receiver Operating Characteristic (ROC) curves, with the Dizziness Handicap Inventory serving as the reference test. The area under the curve (AUC), cutoff points, sensitivity, specificity, and 95% confidence intervals were determined. Statistical analysis was performed using MedCalc® software, with a significance level of *p* < 0.05.

**Results:**

The VAP1-BR showed an AUC of 0.83 (95% CI: 0.744–0.899; *p* < 0.001), a cutoff point ≥9.37, sensitivity of 69%, and specificity of 84%. The VAP2-BR yielded an AUC of 0.80 (95% CI: 0.710–0.875; *p* < 0.001), a cutoff point ≥7.71, sensitivity of 68%, and specificity of 83%.

**Conclusion:**

The VAP subscales adequately identify severe handicap in individuals with vestibular dysfunction.

## Introduction

Vestibular symptoms significantly impact individuals’ quality of life and functionality, as certain activities and environments may trigger symptoms, resulting in activity limitations and participation restrictions.^1–4^ Individuals experiencing vestibular symptoms tend to avoid situations, places, and/or tasks they consider risky or likely to provoke symptoms.^5,6^ This avoidance pattern can contribute to increased disability, since many daily activities may become difficult to perform, ultimately leading to social isolation and impairing the individual’s participation in society.^5,7,8^

To assess how vestibular dysfunction affects quality of life, activity, and participation, instruments such as the Dizziness Handicap Inventory (DHI)^9^ and the Vestibular Activities and Participation Measure (VAP)^10^ subscales are used. Although these instruments assess similar items, the VAP provides a more detailed view of how vestibular dysfunction can result in activity limitation and participation restriction.^11^ These concepts, based on the International Classification of Functioning, Disability and Health (ICF), are essential in healthcare and rehabilitation contexts, as they contribute to understanding the interaction between the patient and their health condition.^11–13^

Self-report instruments can be useful in evaluating functionality in individuals with vestibular dysfunction.^13,14^ Individuals classified with severe handicap by the DHI demonstrate functional deficits on physical examination and self-reported falls, as there is a relationship between the severity of self-perceived disability with dizziness and balance deficits.^15^ For this reason, understanding the severity and impact of vestibular symptoms in the context of activity and participation is important to help healthcare professionals establish specific and personalized intervention plans based on each subject’s health condition.^11^

However, no studies have established sensitivity, specificity, and cutoff points of the VAP for severity of self-perceived handicap. Therefore, the objective of the present study is to evaluate the diagnostic accuracy of the VAP subscales in detecting severe handicap in individuals with vestibular dysfunction.

## Methods

### Study design and setting

This diagnostic accuracy study, using secondary data from the study by Lira et al.,^12^ and followed the Standards for Reporting of Diagnostic Accuracy Studies (STARD) guidelines.^16^

### Ethical considerations

This study was conducted in accordance with the principles of the Declaration of Helsinki and was approved by the research ethics committee of the Federal University of Rio Grande do Norte (no.4,334,272) on 13 of October, 2020.

### Participants

Individuals aged ≥18 years with a medical diagnosis of central and/or peripheral vestibular dysfunction and without cognitive impairment, assessed by the Mini-Mental State Examination (MMSE), were included. Cutoff scores were adjusted according to educational level: 13 for illiterate individuals, 18 for those with elementary or high school education, and 26 for individuals with complete high school or higher education.^17^ Individuals unable to complete any instrument due to comprehension difficulties were excluded.

### Procedures and instruments

Sociodemographic data (age, sex, marital status, education level, and occupation), clinical-functional data (comorbidities, medications, and lifestyle habits such as exercise, alcohol consumption, and smoking), and otoneurological data (vestibular dysfunction diagnosis, presence of tinnitus, aural fullness, oscillopsia, postural imbalance, as well as vertigo, dizziness, and their durations) were collected.

The index test used to assess the disabling effect of dizziness or imbalance on individuals’ activity and participation was the Brazilian version of the Vestibular Activities and Participation Measure (VAP) subscales, previously adapted and validated for the Brazilian population.^12^ The first subscale (VAP1-BR) investigates activities that may trigger vestibular symptoms, while the second subscale (VAP2-BR) evaluates the consequences of these symptoms on activity limitation and participation restriction. Each subscale contains six items with five response options: none = 0, mild = 1, moderate = 2, severe = 3, unable to perform = 4, plus a “not applicable” option. The total score is obtained by summing the item scores of each subscale, where ordinal scores are transformed into interval-scaled scores.^10^

The reference test used to measure self-perceived impact of dizziness in physical, functional, and emotional domains was the Brazilian version of the Dizziness Handicap Inventory (DHI), adapted^18^ and validated for the Brazilian population (r = −0.596).^19^ The DHI includes 25 items, with a total score ranging from 0 to 100 points, calculated by summing the items in each domain.^9^ The total score can be classified as mild disability (0 to 29), moderate disability (30 to 59), and severe disability (60 to 100).^15^

### Statistical analysis

MedCalc® software (version 18.2.1, MedCalc Software Ltd, Ostend, Belgium) was used for statistical analyses. Quantitative variables were described using mean and standard deviation, while categorical variables were presented as absolute and relative frequencies.

The diagnostic accuracy of the VAP subscales’ interval scores to detect severe handicap was measured by the receiver operating characteristic (ROC) curve, using the total DHI score as the reference test. For this analysis, a cutoff point of 60 on the DHI was used to indicate severe handicap, dichotomized as *severe handicap* or *not severe handicap*. Area under the curve (AUC), cutoff point, sensitivity, specificity, positive and negative predictive values, accuracy, and their respective 95% confidence intervals were determined. The discriminative ability of the subscales was classified as outstanding (AUC ≥0.9), excellent (0.8 ≤ AUC <0.9), acceptable (0.7 ≤ AUC <0.8), poor (0.5 < AUC <0.7), or no discrimination (AUC = 0.50).^20^ The statistical significance level was set at p < 0.05.

## Results

The sample consisted of 100 individuals diagnosed with vestibular dysfunction. Separate STARD flowcharts were created for each VAP subscale. The Figure 1 presents both flowcharts, corresponding to the analyses of VAP1-BR (A) and VAP2-BR (B) subscales. In the VAP1-BR subscale, 37 participants tested positive on the index test. As there were no losses, all participants completed the DHI assessment. Of those who tested positive, 24 were classified by the DHI as having the target condition (true positives) and 13 as not having the target condition (false positives). The same interpretation applies to the VAP2-BR (B) flowchart, where 32 participants tested positive on the index test, of whom 22 were classified as having the target condition (true positives) and 10 as not having the target condition (false positives).

**Figure 1.**
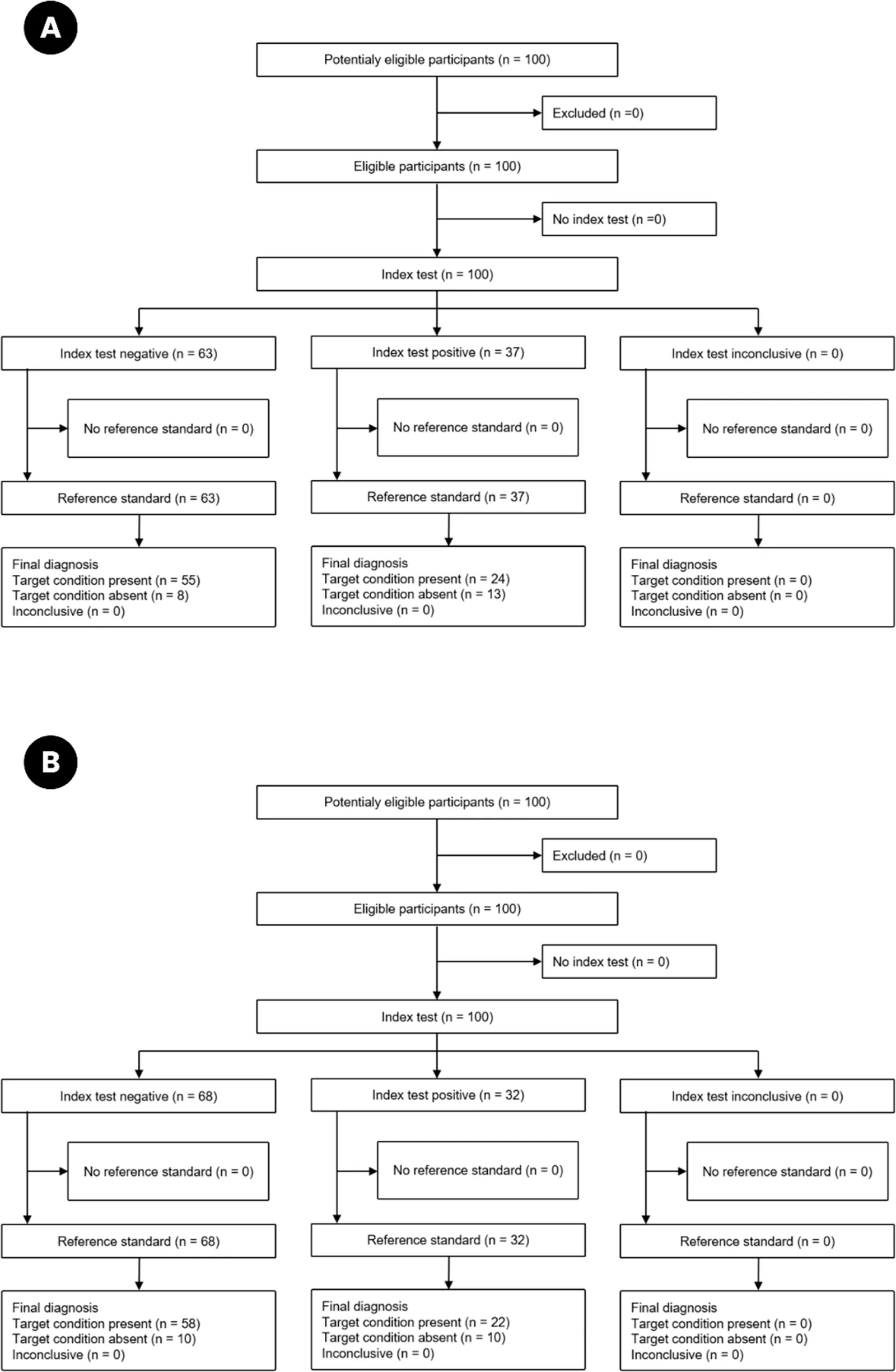
STARD flowcharts for diagnostic accuracy of the VAP1-BR (a) and VAP2-BR (b) subscales (index tests), using the DHI as the reference standard.

The mean age of the sample was 43.4 (±15.1) years, with 80% female. Other sociodemographic and clinical-functional data are presented in Table 1. Peripheral vestibular dysfunction was the most prevalent (46%) diagnostic group, followed by central (35%) and mixed peripheral and central (19%) vestibular disorders. The most frequent diagnoses were vestibular migraine (48%), followed by vestibular hypofunction (23%) and benign paroxysmal positional vertigo (BPPV) (13%). Additional data related to vestibular diagnoses are detailed in Table 2.

**Table 1.** Sample characteristics (*n* = 100).

Variable	Mean (SD)	*n* (%)
Age group		
18–39 years		43 (43)
40–59 years		40 (40)
>60 years		17 (17)
Number of comorbidities	1.5 (1.5)	
Number of medications	1.8 (2.0)	
Sex		
Female		80 (80)
Male		20 (20)
Marital status		
Without partner		48 (48)
With partner		52 (52)
Education level		
Elementary school		7 (7)
High school		30 (30)
Higher education		63 (63)
Occupation		
Employed		56 (56)
Unemployed		44 (44)
Exercise practice		
<3 times/week		9 (9)
>3 times/week		33 (33)
Does not exercise		58 (58)

Data are presented as mean and standard deviation (SD) and as absolute (*n*) and relative (%) frequencies.

**Table 2.** Vestibular diagnoses (*n*, %) of the total sample (*n* = 100).

Vestibular diagnoses	*n* (%)
Peripheral vestibular disorders	
Vestibular hypofunction	23 (23)
BPPV	13 (13)
Meniere’s disease	4 (4)
Vestibular neuritis	2 (2)
Meniere’s disease + vestibular hypofunction	1 (1)
BPPV + vestibular hypofunction	1 (1)
Cervicogenic dizziness	1 (1)
Elderly imbalance syndrome	1 (1)
Central vestibular disorders	
Vestibular migraine	29 (29)
Vasovagal syndrome	2 (2)
Cerebellar stroke	1 (1)
Arnold-Chiari disease	1 (1)
Parkinson’s disease	1 (1)
Cerebral palsy	1 (1)
Mixed (central and peripheral)	
Vestibular migraine + vestibular hypofunction	13 (13)
Vestibular migraine + BPPV	4 (4)
Vestibular migraine + vestibular hypofunction + BPPV	1 (I)
Vestibular migraine + Meniere’s disease	1 (1)

Data are presented as absolute (*n*) and relative (%) frequencies.

Figure 2 shows the ROC curves of the VAP1-BR (A) and VAP2-BR (B) subscales. The VAP1-BR subscale presented an AUC of 0.83 (95% CI: 0.744–0.899; *p* < 0.001), whereas the VAP2-BR obtained an AUC of 0.80 (95% CI: 0.710–0.875; *p* < 0.001). As shown in Table 3, the VAP1-BR subscale classified 24 individuals as true positives (TPs) and 55 as true negatives (TNs). For the VAP2-BR subscale, 22 individuals were classified as TPs and 58 as TNs.

**Figure 2.**
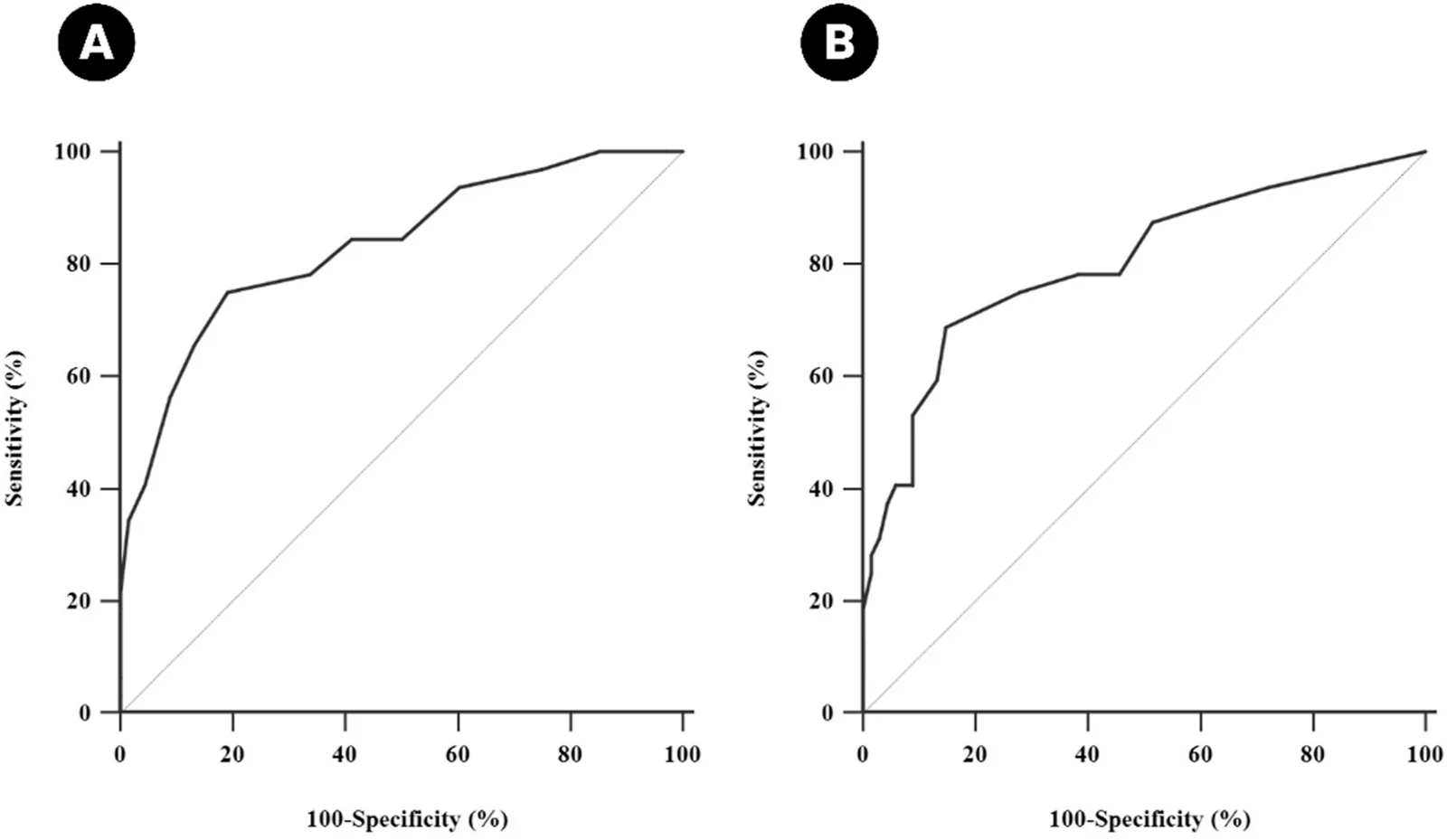
Area under the ROC curve for detecting severe handicap in individuals with vestibular dysfunction: (a) VAP1-BR and (b) VAP2-BR.

**Table 3.** Quantitative analysis of severe (DHI ≥60) and not severe handicap (≤59) detected by the DHI, VAP1-BR, and VAP2-BR.

	DHI		
	Not severe handicap	Severe handicap	Total
VAP1-BR			
Severe handicap^a^	13 (FP)	24 (TP)	37
Not severe handicap	55 (TN)	8 (FN)	63
**Total**	68	32	100
VAP2-BR			
Severe handicap^b^	10 (FP)	22 (TP)	32
Not severe handicap	58 (TN)	10 (FN)	68
**Total**	68	32	100

FP: false positive; TP: true positive; TN: true negative; FN: false negative.

^a^Cutoff point of ≥9.37.

^b^Cutoff point of ≥7.71.

Table 4 presents the values of sensitivity, specificity, positive and negative predictive values, and accuracy along with their respective 95% confidence intervals. The cutoff point for severe handicap was ≥9.37 for VAP1-BR and ≥7.71 for VAP2-BR.

**Table 4.** Sensitivity, specificity, positive predictive value, negative predictive value, and accuracy of the VAP1-BR and VAP2-BR for severe handicap screening in people with vestibular disorders.

	VAP1-BR	VAP2-BR
Sensitivity %	75.0 (56.6 to 88.5)	68.7 (50.0 to 83.9)
Specificity %	80.9 (68.5 to 89.4)	85.3 (74.6 to 92.7)
Positive predictive value %	68.7 (54.2 to 80.3)	64.9 (52.1 to 75.8)
Negative predictive value %	85.3 (77.5 to 90.7)	87.3 (78.9 to 92.7)
Accuracy %	80.0 (70.8 to 87.3)	79.0 (69.7 to 86.5)

Values are presented as percentages with 95% confidence intervals.

## Discussion

This study aimed to evaluate the diagnostic accuracy of the VAP subscales in detecting severe handicap in individuals with vestibular dysfunction. The results demonstrated that the VAP subscales can adequately identify severe handicap in activity and participation. Defining cutoff points for the VAP subscales makes them useful in clinical practice, facilitating decision-making.^21^

A systematic review on instruments used in otology identified 23 questionnaires related to vertigo, 33 to tinnitus, and 84 to hearing loss, highlighting the role of Patient-Reported Outcome Measurements (PROMs) in assessing subjective complaints and disability in patients with otologic conditions.^22^ Among these instruments, the DHI is a widely used PROM for evaluating the impact of dizziness in functional, physical, and emotional domains.^9^ Its cutoff points are recognized worldwide^23–25^ and allow for the identification of varying levels of handicap, including severe cases.^26^ Total scores above 60 on the DHI have been associated with greater functional impairment, reduced confidence in both static and dynamic balance, and an increased risk of falls.^15^ Additionally, individuals with DHI scores ≤30 are commonly associated with structural disorders (specificity = 98%), while scores >60 are typically linked to functional or psychiatric conditions (sensitivity = 83%), with or without coexisting structural problems.^27^ For this reason, the present study adopted this cutoff point as a reference for analyzing the diagnostic accuracy of the VAP subscales in identifying severe handicap. Furthermore, due to its widespread use in both clinical practice and research,^23–25,28,29^ the DHI has been used as a reference test for construct validation, given its similarity to the items in the VAP subscales, as seen in studies by Mueller et al.,^10^ Wu et al.,^30^ and Lira et al.^12^

Sharda et al.^31^ investigated the association between the items of the DHI and the original version of the VAP scale and found that less than half of the DHI items showed strong or moderate associations with the total VAP score. These findings suggest that a small portion of DHI items reflect activity limitations or participation restrictions in individuals with vestibular dysfunction, emphasizing that the VAP is a more suitable instrument for specifically activity and participation, thereby allowing a more focused analysis of functional outcomes in daily life.^30,31^ Moreover, the VAP subscales encompass fewer items compared to the DHI,^18^ favoring a quicker assessment of activity and participation in clinical practice.^11^ Identifying functional limitations in individuals with vestibular dysfunction is essential, as these are among the primary targets of physical therapy interventions and are directly related to the patient’s functional capacity.^6,10,32^ Thus, tailored interventions for individuals with vestibular symptoms contribute to improvements in both activity and participation outcomes.^10,11^

The VAP subscales have different scores due to disordered thresholds in the response levels, as identified by Mueller et al.^10^ through Rasch analysis, indicating a non-linear relationship with the individual’s ability. To correct this inconsistency, the response levels were adjusted, reducing the score for item 6 of the VAP1-BR, adjusted to a total score of 23 points, and for items 2, 3, and 6 of the VAP2-BR, arranged to a total score of 20 points.^10^ Based on these changes, our study established distinct cutoff points for each subscale, with a lower cutoff for the VAP2-BR to avoid overclassification of severe handicap. This subscale involves more demanding tasks than the VAP1-BR, particularly those related to mobility, in which even mild impairments may indicate relevant functional limitations.

The AUC results from the integration of points corresponding to sensitivity and specificity along the ROC curve, providing the test’s accuracy, which corresponds to an estimate of the probability of correctly classifying a health condition.^33^ In this context, the AUC values obtained for the two VAP-BR subscales were considered excellent,^20^ indicating the ability to discriminate individuals with severe handicap among those with vestibular dysfunction. In clinical settings, accurate, reliable, and objective outcome measures can facilitate screening by level of handicap and standardization of assessment among different professionals.^10,34^ The use of an instrument with well-established cutoff points supports clinical decision-making and helps in selecting interventions most appropriate to the patient’s health condition, contributing to a better prognosis.^11,21^

Some limitations of this study should be considered. The lack of a sample size calculation may have compromised the representativeness of the sample. In addition, the small number of participants over 60 years of age and with low educational levels may limit the generalizability of the results to older or less-educated populations. Furthermore, the sample had a high proportion of female participants, which may affect the external validity of the findings. Finally, the low number of individuals with Benign Paroxysmal Positional Vertigo (BPPV) should be noted, likely because assessments were conducted remotely during the coronavirus disease 2019 (COVID-19) pandemic, which may have limited the identification of BPPV cases. Among the strengths of this study is the fact that it is the first to propose cutoff points for the VAP subscales using a robust statistical method (ROC curves). Furthermore, the sample consisted of clinically diagnosed individuals and employed validated instruments, which strengthens the methodological rigor of the study and broadens the potential applicability of the results in clinical practice.

## Conclusion

The VAP subscales demonstrated adequate diagnostic accuracy in identifying severe handicap in individuals with vestibular dysfunction, with cutoff points of ≥9.37 for the VAP1-BR and ≥7.71 for the VAP2-BR. In addition to their applicability for clinical screening, the VAP subscales may assist healthcare professionals in the development and implementation of personalized therapeutic plans, promoting more effective care for people at higher risk.

## Data Availability

The datasets generated during and/or analyzed during the current study are not publicly available due restrictions imposed by the ethics committee but are available from the corresponding author on reasonable request.*
